# Supplementary material for: Evidence of emotion dysregulation as a core symptom of adult ADHD: A systematic review
Source: PLoS One. 2023 Jan 6;18(1):e0280131. doi: 10.1371/journal.pone.0280131 (PMC9821724; doi:10.1371/journal.pone.0280131)
Supplement: S1 Table — (DOCX) [file pone.0280131.s001.docx]

| **Title** | **Authors** | **Year** | **Journal, number, and pages** | **Metrics (SJR, 2021)** | **Scopus citations** |
| --- | --- | --- | --- | --- | --- |
| Prevalence of criminal convictions in Norwegian adult ADHD outpatients and associations with ADHD symptom severity and emotional dysregulation. | Anker et al. [67] | 2021 | BMC Psychiatry, 21:226 | **1.252 SJR (H index: 112). Country:** UK**. Subject area-Category:**  Medicine – Psychiatry and Mental Health. **Quartile: Q1** | 0 |
| Alcohol and drug use disorders in adult attention deficit/ hyperactivity disorder: Prevalence and associations with attention-deficit/ hyperactivity disorder symptom severity and emotional dysregulation. | Anker et al. [64] | 2020 | World J Psychiatr, 10(9), 202-211 | ***4.571 JCR (Journal citation indicator: 0.73). Country:** USA**. Subject area-Category:** Psychiatry-SCIE**. Quartile: Q2** | *6 |
| Mentalization-based treatment for adults with attention-deficit/hyperactivity disorder: a pilot study. | Badoud et al. [72] | 2018 | Research in Psychotherapy: Psychopathology, Process and Outcome, 21, 149-154 | **0.379 SJR (H index: 14).** **Country**: Italy. **Subject area-Category:** Medicine – Psychiatry and Mental Health; Psychology – Clinical Psychology. **Quartile: Q3/Q3** | 1 |
| Comparison of Emotional Dysregulation Features in Cyclothymia and Adult ADHD. | Brancati et al. [68] | 2021 | Medicina, 57, 489 | **0.536 SJR (H index: 42).**  **Country:** Switzerland**. Subject area-Category:** Medicine – Miscellaneous. **Quartile: Q2** | 0 |
| A comparison of self-reported emotional regulation skills in adults with attention- deficit/hyperactivity disorder and borderline personality disorder. | Cavelti et al. [58] | 2019 | Journal of Attention Disorders, 23(12), 1396-1406 | **1.066 SJR (H index: 76). Country:** US**. Subject area-Category:** Psychology – Clinical Psychology, Developmental and Educational Psychology**. Quartile: Q1/Q1** | 12 |
| Interaction of recalled parental ADHD symptoms and rearing behaviour with current attachment and emotional dysfunction in adult offspring with ADHD. | Edel et al. [65] | 2010 | Psychiatry Research, 178, 137–141 | **1.656 SJR (H index: 147).** **Country**: Ireland. **Subject area-Category:** Medicine – Psychiatry and Mental Health; Neuroscience – Biological Psychiatry. **Quartile: Q1/Q1** | 13 |
| Emotion recognition and mind wandering in adults with attention deficit hyperactivity disorder or autism spectrum disorder. | Helfer et al. [59] | 2021 | Journal of Psychiatric Research, 134, 89-96 | **1.56 SJR (H index: 144). Country:** UK. **Subject area-Category**: Medicine – Psychiatry and Mental Health; Neuroscience – Biological Psychiatry. **Quartile: Q1/Q1** | 2 |
| Emotional dysregulation subgroups in patients with adult Attention- Deficit/Hyperactivity Disorder (ADHD): a cluster analytic approach. | Hirsch et al. [60] | 2019 | Scientific Reports, 9(1), 5639 | **1.005 SJR (H index: 242).** **Country**: UK. **Subject area-Category:** Multidisciplinary. **Quartile: Q1.** | 15 |
| Emotional dysregulation is a primary symptom in adult Attention-Deficit/Hyperactivity Disorder (ADHD). | Hirsch et al. [13] | 2018 | Journal of Affective Disorders, 232, 41–47 | **1.791 SJR (H index: 205).** **Country**: Netherlands. **Subject area-Category:** Medicine – Psychiatry and Mental Health; Psychology – Clinical Psychology. **Quartile: Q1/Q1** | 55 |
| Increased Beta Activity Links to Impaired Emotional Control in ADHD Adults with High IQ. | Li et al. [54] | 2017 | Journal of Attention Disorders 23(7), 754-764 | **1.066 SJR (H index: 76). Country:** US**. Subject area-Category:** Psychology – Clinical Psychology, Developmental and Educational Psychology**. Quartile: Q1/Q1** | 5 |
| Adult patients with ADHD differ from healthy controls in implicit, but not explicit, emotion regulation. | Materna et al. [51] | 2019 | J Psychiatry Neurosci, 44(5), 340-349 | **1.474 SJR (H index: 104).** **Country**: Canada. **Subject area-Category:** Medicine – Miscellaneous, Pharmacology, Psychiatry and Mental Health; Neuroscience –Biological Psychiatry. **Quartile: Q1/Q1/Q1/Q1** | 8 |
| Regulation of sadness via acceptance or suppression in adult Attention Deficit Hyperactivity Disorder (ADHD). | Matthies et al. [69] | 2014 | Psychiatry Research, 220, 461–467 | **1.656 SJR (H index: 147).** **Country**: Ireland. **Subject area-Category:** Medicine – Psychiatry and Mental Health; Neuroscience – Biological Psychiatry. **Quartile: Q1/Q1** | 9 |
| Smoking abstinence effects on emotion dysregulation in adult cigarette smokers with and without attention-deficit/ hyperactivity disorder. | Mitchell et al. [61] | 2019 | Drug and Alcohol Dependence, 205, 107594 | **1.438 SJR (H index: 173).** **Country**: Ireland. **Subject area-Category:** Medicine – Pharmacology, Psychiatry and Mental Health; Pharmacology, Toxicology y Pharmaceutics – Pharmacology, Toxicology. **Quartile: Q1/Q1/Q1/Q1** | 1 |
| A pilot trial of mindfulness meditation training for ADHD in adulthood: impact on core symptoms, executive functioning, and emotion dysregulation. | Mitchell et al. [71] | 2017 | Journal of Attention Disorders 21(13), 1105-1120 | **1.066 SJR (H index: 76). Country:** US**. Subject area-Category:** Psychology – Clinical Psychology, Developmental and Educational Psychology**. Quartile: Q1/Q1** | 90 |
| Comparable emotional dynamics in women with ADHD and borderline personality disorder. | Moukhtarian et al. [62] | 2021 | Borderline Personality Disorder and Emotion Dysregulation 8(1),6 | **1.243 SJR (H index: 21).** **Country**: UK. **Subject area-Category:** Medicine – Psychiatry and Mental Health; Neuroscience – Biological Psychiatry; Psychology – Clinical Psychology. **Quartile: Q1/Q2/Q1** | 1 |
| Emotional Dysregulation in Adult ADHD and Response to Atomoxetine. | Reimherr et al. [73] | 2005 | Biological Psychiatry  58(2), 125-131 | **4.224 SJR (H index: 333).** **Country**: USA. **Subject area-Category:** Neuroscience – Biological Psychiatry. **Quartile: Q1** | 169 |
| Double-blind, placebo-controlled, crossover study of osmotic release oral system methylphenidate in adults with ADHD with assessment of oppositional and emotional dimensions of the disorder. | Reimherr et al. [74] | 2007 | Journal of Clinical Psychiatry  68(1), 93-101 | **1.362 SJR (H index: 212).** **Country**: USA. **Subject area-Category:** Medicine – Miscellaneous, Psychiatry and Mental Health. **Quartile: Q1/Q1** | 143 |
| Emotion dysregulation in adults suffering from attention deficit hyperactivity disorder (ADHD), a comparison with borderline personality disorder (BPD). | Rüfenacht et al. [63] | 2016 | Borderline Personality Disorder and Emotion Dysregulation, 6(11) | **1.243 SJR (H index: 21).** **Country**: UK. **Subject area-Category:** Medicine – Psychiatry and Mental Health; Neuroscience – Biological Psychiatry; Psychology – Clinical Psychology. **Quartile: Q1/Q2/Q1** | 10 |
| Exploring deficient emotion regulation in adult ADHD: electrophysiological evidence. | Shushakova et al. [52] | 2018 | Eur Arch Psychiatry Clin Neurosci 268, 359–371 | **1.222 SJR (H index: 102).** **Country**: Germany. **Subject area-Category:** Medicine – Miscellaneous, Pharmacology, Psychiatry and Mental Health; Neuroscience – Biological Psychiatry. **Quartile: Q1/Q1/Q1/Q2** | 14 |
| Electrophysiological evidence of an attentional bias towards appetitive and aversive words in adults with attention-deficit/hyperactivity disorder. | Shushakova et al. [70] | 2018 | Clinical Neurophysiology 129, 1937–1946 | **1.0638 SJR (H index: 191).** **Country**: Ireland. **Subject area-Category:** Medicine – Neurology; Physiology; Neuroscience – Neurology; Sensorial Systems. **Quartile: Q1/Q2/Q1/Q1** | 5 |
| The Characteristics and Unique Impairments of Comorbid Adult ADHD and Sluggish Cognitive Tempo: An Interim Analysis | Silverstein et al. [66] | 2019 | Psychiatric Annals  49(10), 457-465 | **0.201 SJR (H index: 39).** **Country**: USA. **Subject area-Category:** Medicine – Psychiatry and Mental Health. **Quartile: Q4** | 2 |
| Emotion dysregulation in adult ADHD: Introducing the Comprehensive Emotion Regulation Inventory (CERI) | Thorell et al. [53] | 2020 | Journal of Clinical and Experimental Neuropsychology  42(7), 747-758 | **0.568 SJR (H index: 113).** **Country**: UK. **Subject area-Category:** Medicine – Neurology (clinical); Neuroscience – Neurology; Psychology – Clinical Psychology, Neuropsychology and Physiological Psychology. **Quartile: Q3/Q3/Q2/Q3** | 1 |

*Data from Journal Citation Reports (JCR), and Web of Science (WOS)
