# Supplementary material for: Evidence of emotion dysregulation as a core symptom of adult ADHD: A systematic review
Source: PLoS One. 2023 Jan 6;18(1):e0280131. doi: 10.1371/journal.pone.0280131 (PMC9821724; doi:10.1371/journal.pone.0280131)
Supplement: S2 Table — (DOCX) [file pone.0280131.s002.docx]

| **Author** | **Country** | **Sample origin Cases/Controls** | **Sample size (n) Cases/Controls** | **Age (M/SD)**  **Cases/Controls** | **Sex (% M) Cases/Controls** | **Subtype TDAH**  **I / C (%)** | **Comorbidity Cases (%)** | **ADHD Medication**  **status (%)** |
| --- | --- | --- | --- | --- | --- | --- | --- | --- |
| Anker et al. [67] | Norway | Clinical | 629/0 | 36.7 (11.4) | 53.9 | - | - | 0 |
| Anker et al. [64] | Norway | Clinical | 585/0 | 36.8 (11.4) | 54.2 | - | 16.4 | 0 |
| Badoud et al. [72] | Switzerland | Clinical | 8/0 | 43.25 (12.8) | 25 | 25/75 | 0 | 100 |
| Brancati et al. [68] | Italy | Clinical | 80/0 | 27.88 (9.69) | 69.5 | - | - | - |
| Cavelti et al. [58] | Switzerland | Clinical/ no-Clinical | 80/55 | 33.39 (9.4)/  33.33 (11.56) | 62.5/54.5 | - | - | - |
| Edel et al. [65] | Germany | Clinical | 73/0 | 40 (9.7) | 53.4 | 46.5/53.5 | - | 72.2 |
| Helfer et al. [59] | UK | Clinical/ no-Clinical | 43/46 | 37.16 (10.06)/ 29.37 (9.06) | 62.8/43.5 | - | 0 | - |
| Hirsch et al. [60] | Germany | Clinical | 385/0 | 32.45 (10) | 62.9 | - | 47.4 | 0 |
| Hirsch et al. [13] | Germany | Clinical | 213/0 | 33.25 (10.45) | 60.5 | - | 48.3 | 0 |
| Li et al. [54] | China | Clinical/ no- Clinical | 40/40 | 25.85 (5.21)/ 25.88 (3.83) | 57.5/65 | 72.5/22.5 | 22.5 | 0 |
| Materna et al. [51] | Germany | Clinical/ no-Clinical | 30/35 | 31.40 (8.21)/  28.89 (7.77) | 63/54 | 33.3/66.7 | 13 | 30 |
| Matthies et al. [69] | Germany | Clinical | 36/0 | 37.37 (11.33) | 47.2 | - | 88.9 | - |
| Mitchell et al. [61] | USA | No Clinical | 19/20 | 33.32 (9.54)/  30.70 (8.20) | 53/40 | 57.9/42.1 | - | - |
| Mitchell et al. [71] | USA | Mixed / no Clinical | 11/9 | 40.55 (6.83)/  36.22 (6.92) | 40 | 70/30 | 40 | 55 |
| Moukhtarian et al. [62] | UK | Clinical/ no-Clinical | 28/29 | 38.2 (11.7)/ 27.1 (5.2) | 0/0 | - | - | - |
| Reimherr et al. [73] | USA/Canada | Clinical | 536/0 | 41.2 (11.2) | 65 | 31/66 | 0 | 45 |
| Reimherr et al. [74] | USA | Clinical | 47/0 | 30.6 (10.8) | 66 | - | 0 | - |
| Rüfenacht et al. [63] | Switzerland | Clinical | 279/0 | 35.49 (12.86) | 56 | 39/56.6 | - | 12.9 |
| Shushakova et al. [52] | Germany | Clinical/ no-Clinical | 39/40 | 31.21 (8.27)/  31.08 (8.83) | 53.8/55 | 46.2/53.8 | 10.3 | 35.9 |
| Shushakova et al. [70] | Germany | Clinical/ no-Clinical | 39/41 | 31.15 (8.24)/  30.59 (8.95) | 53.8/58.5 | 43.6/56.4 | 10.3 | 35.9 |
| Silverstein et al. [66] | USA | Clinical | 87 | 32.4 (6.9) | 33.3 | - | 10.3 | 0 |
| Thorell et al. [53] | Switzerland | Clinical/ no-Clinical | 130/260 | 33.11 (9.93) / 37.3 (7.77) | 33.1/30.8 | 15.4/85.4 | 30 | - |

* These studies have other clinical groups. Only ADHD and Healthy Control groups have been selected.
