# Supplementary material for: Evidence of emotion dysregulation as a core symptom of adult ADHD: A systematic review
Source: PLoS One. 2023 Jan 6;18(1):e0280131. doi: 10.1371/journal.pone.0280131 (PMC9821724; doi:10.1371/journal.pone.0280131)
Supplement: Attachment — Submitted filename: Response to Reviewers.docx [file pone.0280131.s005.docx]

Responses to Journal requirements:

When submitting your revision, we need you to address these additional requirements.

1. Please ensure that your manuscript meets PLOS ONE's style requirements, including those for file naming.

*We have check that all files meet PLOS ONE’s requirements.*

2. Thank you for stating the following financial disclosure:

This work was supported by Universidad Nacional de Educación a Distancia (UNED) through an FPI-UNED grant given to A.M.S.G.

Please state what role the funders took in the study.  If the funders had no role, please state: "The funders had no role in study design, data collection and analysis, decision to publish, or preparation of the manuscript." 
If this statement is not correct you must amend it as needed. 
Please include this amended Role of Funder statement in your cover letter; we will change the online submission form on your behalf.

*In our reviewed submission we have stated that the funders had no role in study design, data collection and analysis, decision to publish, or preparation of the manuscript, as indicated in our cover letter.*

3. In your Data Availability statement, you have not specified where the minimal data set underlying the results described in your manuscript can be found. PLOS defines a study's minimal data set as the underlying data used to reach the conclusions drawn in the manuscript and any additional data required to replicate the reported study findings in their entirety. All PLOS journals require that the minimal data set be made fully available. For more information about our data policy, please see http://journals.plos.org/plosone/s/data-availability.

"Upon re-submitting your revised manuscript, please upload your study’s minimal underlying data set as either Supporting Information files or to a stable, public repository and include the relevant URLs, DOIs, or accession numbers within your revised cover letter. For a list of acceptable repositories, please see http://journals.plos.org/plosone/s/data-availability#loc-recommended-repositories. Any potentially identifying patient information must be fully anonymized.

Important: If there are ethical or legal restrictions to sharing your data publicly, please explain these restrictions in detail. Please see our guidelines for more information on what we consider unacceptable restrictions to publicly sharing data: http://journals.plos.org/plosone/s/data-availability#loc-unacceptable-data-access-restrictions. Note that it is not acceptable for the authors to be the sole named individuals responsible for ensuring data access.

We will update your Data Availability statement to reflect the information you provide in your cover letter.

*We have attached our research dataset as a Supporting Information excel file. We state in our cover letter that our dataset is fully available.*

4. PLOS requires an ORCID iD for the corresponding author in Editorial Manager on papers submitted after December 6th, 2016. Please ensure that you have an ORCID iD and that it is validated in Editorial Manager. To do this, go to ‘Update my Information’ (in the upper left-hand corner of the main menu), and click on the Fetch/Validate link next to the ORCID field. This will take you to the ORCID site and allow you to create a new iD or authenticate a pre-existing iD in Editorial Manager. Please see the following video for instructions on linking an ORCID iD to your Editorial Manager account: https://www.youtube.com/watch?v=_xcclfuvtxQ

*Correspondence author has validated her ORCID iD in Editorial Manager.*

5. Please include captions for your Supporting Information files at the end of your manuscript, and update any in-text citations to match accordingly. Please see our Supporting Information guidelines for more information: http://journals.plos.org/plosone/s/supporting-information.

*We have included captions for our Supporting Information files at the end of our manuscript and update in-text citations accordingly.*

6. Please review your reference list to ensure that it is complete and correct. If you have cited papers that have been retracted, please include the rationale for doing so in the manuscript text, or remove these references and replace them with relevant current references. Any changes to the reference list should be mentioned in the rebuttal letter that accompanies your revised manuscript. If you need to cite a retracted article, indicate the article’s retracted status in the References list and also include a citation and full reference for the retraction notice.

*We have included two news references [108, 109] following the recommendations of our reviewer*.

Responses to the Reviewer's comments:

Reviewer #1: I would like to congratulate the authors on this interesting and clearly written systematic literature review. Emotion dysregulation in adults with ADHD is a timely topic and a review would be a welcome contribution to the existing literature. The introduction provides a solid background of the field with recent references. The results are well organized and supported by exhaustive tables. The discussion section summarizes the main findings and conclusions, which are related back to the set hypotheses.

*Thank you very much for your kind feedback. We greatly appreciate the suggested improvements and invitation to submit a revised version of our manuscript.*

My main point of feedback is about the discussion section: it could benefit from a more critical discussion of the limitations and presentation of a research agenda. For example, the review identified several factors that need to be taken into account when studying emotion regulation in ADHD, such as sex differences, medication use, different types of emotion regulation strategies, overlap with other disorders, etc. It would be very helpful to field if the most important factors would be discussed, so to foster high quality research in this field. Perhaps the authors could think of a research agenda?

*Firstly, thank you for bringing up this point. We have followed this recommendation discussing three main factors that future research need to consider: sex differences, medication status and pre-existing comorbidities, as they could get conflicting outcomes.*

One important point that needs consideration is the specificity of emotion regulation problems for ADHD; it is stated (p.21, line 337) that emotion dysregulation are core components of ADHD. However, this conclusion may be too preliminary, since also other disorders are characterized by these impairments, e.g. autism, anxiety, depression. In order to conclude that emotion dysregulation is part of the disorder, more studies need to be carried out to determine the specificity for ADHD and also about the causes of these problems. For example, it is mentioned that these problems are the result of top-down processes and that emotion regulation improves when top-down processes improve. Emotion dysregulation may therefore be secondary to cognitive dysregulation. The authors might be interested in a recent model of regulation for applied settings by Bailey & Jones (2019) https://doi.org/10.1007/s10567-019-00288-y, showing that emotion regulation needs to be integrated in research with cognitive as well as social regulation. Emotion regulation is just one aspect of self-regulation in humans and may covary with the other domains, in the case of ADHD most likely the cognitive domain.

*We totally agree with this suggestion. The evidence obtained to date does not allow us to ensure that ED is an ADHD core symptom, so we have made it clear. Thank you for such a useful reference, so it fits with our research and help to explain our conclusions. That is why we have included it.*

Furthermore, some methodological limitations could be discussed, such as the use of self-reports which could be less reliable in adults with ADHD because of over- or underestimation of their own symptoms on retrospective questionnaires, e.g. Wallace et al., 2019, https://doi.org/10.1037/pas0000659 . It may be necessary to use more objective or ecological momentary assessments for emotion regulation in future studies.

*Certainly, the diagnostic process for ADHD needs to include more objective and reliable tests. We have taken into account the suggested reference to improve our discussion on this issue.*

Below I formulated several minor errors and irregularities I found in the paper:
Abstract: nuclear symptom is a strange wording -> core symptom

*We have changed* nuclear *for the better one* core

Whole paper: sometimes the word emotional (dys)regulation is used -> use consistently emotion (dys)regulation;

*We made changes needed to use consistently emotion dysregulation. However, in some cases was not appropriate since reference is made to the global process of emotion regulation, so we maintain the term emotion regulation in these occasions.*

sometimes the word subjects is used to describe the participants, which is a disrespectful term for the participants and should be avoided according to APA style.

*Thank you for letting us know about this issue. We have replaced subjects for participants.*

Introduction: the aims could be written more concise by coupling aim and hypotheses, less enters need to be used.

*We have reformulated this point to make it more concise.*

Method: p.7 it says that participants over age 18 were included, but the flow-chart says over 16 – which is the correct? In the procedure section it needs mentioning why no meta-analysis was conducted – this only comes back in the discussion.

*Certainly, it was an error in age. We have corrected it in the flowchart. In addition, we have clarified the reason for not carrying out meta-analyses*

Tables 1&2 could be added to the supplements, because they only provides background information.

*Following this suggestion, we have added tables 1 and 2 as supporting information and made the necessary adjustments to the main text.*

Results: p.15 line 206 was the ER score consistently lower? How many studies did not find this effect? P.16 line 216 variance of what? Regarding the treatment studies: clear up if the outcome measures were blinded/ who the rater was.

*We have indicated in the text the number of studies that found the commented results, also in discussion section. Regarding treatments, we have clarified if they were controlled and/or blinded, but in three of them we are not clear the measures of ED are self-reported or observed by the clinician.*

Discussion: p.21 line 335 it says that 34-70% of adults with ADHD are affected by emotion dysregulation, but it is unclear whether this stems from the review results or just one study. It is a bit confusing in this first paragraph whether these are conclusions from the review or not. When discussing the findings it would be helpful to mention the number/percentage of studies that found positive or negative results to get an idea of the consistency of the findings. I am not sure whether the conclusion that emotion dysregulation is a core component of ADHD is sufficiently supported by the review and current research (see above).

*We have reformulated the first discussion paragraph to clarify this issue, since it is a conclusion of other authors. Also explaining the controversy of whether ED is a core symptom of ADHD. Throughout the section, we have indicated the number of articles that obtain the mentioned results.*

A final concluding paragraph would be helpful.

*Finally, we have made some changes in the last part of the discussion, writing the last paragraph as a conclusion.*

6. PLOS authors have the option to publish the peer review history of their article ([what does this mean?](https://journals.plos.org/plosone/s/editorial-and-peer-review-process#loc-peer-review-history)). If published, this will include your full peer review and any attached files.


If you choose “no”, your identity will remain anonymous but your review may still be made public.


**Do you want your identity to be public for this peer review?** For information about this choice, including consent withdrawal, please see our [Privacy Policy](https://www.plos.org/privacy-policy).

Reviewer #1: **Yes:** Dr. Yvonne Groen

[NOTE: If reviewer comments were submitted as an attachment file, they will be attached to this email and accessible via the submission site. Please log into your account, locate the manuscript record, and check for the action link "View Attachments". If this link does not appear, there are no attachment files.]

While revising your submission, please upload your figure files to the Preflight Analysis and Conversion Engine (PACE) digital diagnostic tool, <https://pacev2.apexcovantage.com/>. PACE helps ensure that figures meet PLOS requirements. To use PACE, you must first register as a user. Registration is free. Then, login and navigate to the UPLOAD tab, where you will find detailed instructions on how to use the tool. If you encounter any issues or have any questions when using PACE, please email PLOS at [figures@plos.org](mailto:figures@plos.org). Please note that Supporting Information files do not need this step.
